# The microbiota of Mozzarella di Bufala Campana PDO cheese: a study across the manufacturing process

**DOI:** 10.3389/fmicb.2023.1196879

**Published:** 2023-08-15

**Authors:** Alessia Levante, Gaia Bertani, Martina Marrella, Germano Mucchetti, Valentina Bernini, Camilla Lazzi, Erasmo Neviani

**Affiliations:** Department of Food and Drug, University of Parma, Parma, Italy

**Keywords:** Mozzarella di Bufala Campana PDO, lactic acid bacteria, natural whey starter, *Lactobacillus* spp., *Streptococcus* spp., 16S rRNA amplicon sequencing, fermented food, microbial community

## Abstract

**Introduction:**

Mozzarella di Bufala Campana PDO cheese (MBC) is a globally esteemed Italian cheese. The traditional cheesemaking process of MBC relies on natural whey starter culture, water buffalo's milk, and the local agroecosystem.

**Methods:**

In this study, the microbial ecology of intermediate samples of MBC production, coming from two dairies with slightly different cheesemaking technology (dairy M large producer, and dairy C medium-small), was investigated using 16S rRNA amplicon sequencing. This research aimed to provide insights into the dynamics of microbial consortia involved in various cheesemaking steps.

**Results and discussion:**

All samples, except for raw buffalo milk, exhibited a core microbiome predominantly composed of *Streptococcus* spp. and *Lactobacillus* spp., albeit with different ratios between the two genera across the two MBC producers. Notably, the microbiota of the brine from both dairies, analyzed using 16S amplicon sequencing for the first time, was dominated by the *Lactobacillus* and *Streptococcus* genera, while only dairy C showed the presence of minor genera such as *Pediococcus* and *Lentilactobacillus*. Intriguingly, the final mozzarella samples from both producers displayed an inversion in the dominance of *Lactobacillus* spp. over *Streptococcus* spp. in the microbiota compared to curd samples, possibly attributable to the alleviation of thermal stress following the curd stretching step. In conclusion, the different samples from the two production facilities did not exhibit significant differences in terms of the species involved in MBC cheesemaking. This finding confirms that the key role in the MBC cheesemaking process lies with a small-sized microbiome primarily composed of *Streptococcus* and *Lactobacillus* spp.

## 1. Introduction

Several traditional dairy products have obtained the Protected Designation of Origin (PDO) recognition under the European Union regulation, yet Mozzarella di Bufala Campana (MBC) was the first mozzarella-type cheese to gain this recognition back in June 1996 [Commission Regulation (EC) No 1107/96], which was subsequently amended regarding the extension of the geographical area (N. CE: IT/PDO/117/0014/04.02.2008). This fresh “pasta filata” cheese, unique for its low salt content, shape, color, and stretchability is manufactured in Campania, especially in the provinces of Caserta and Salerno. MBC cheesemaking starts with raw or pasteurized water buffalo milk brought to a temperature of ~35–37°C and subsequently inoculated with natural whey starter (NWS) culture and calf rennet. During the curd-ripening phase (4–4.5 h at 35–37°C), only when it has reached the optimal pH of ~4.9–4.8, the mature curd, under hot (boiling) water of ~90–95°C, becomes stretchable. The elastic product obtained is finally molded to reach the final round shape typical of MBC cheese, hardened in fresh water, and salted by brining (Gobbetti et al., 2018; Fusco et al., 2022).

The association and the combination between the NWS microbial composition, together with the water buffalo's milk, the agroecosystem, and the traditional manufacturing technology, shape and drive the technological process of MBC cheesemaking, leading to one of the most known and appreciated cheeses worldwide (Ercolini et al., 2012; Marino et al., 2019).

NWS culture is of common use in many Italian and European long-ripened cheese varieties, such as Grana Padano, Parmigiano Reggiano, Comté, and Gruyère. It is a complex and variable microbial *consortium*, characterized by multiple-strain cultures of thermophilic lactic acid bacteria (LAB), as well as minority mesophilic bacteria, and its preparation through a traditional back-slopping procedure establishes a microbiological *liaison* among subsequent batches of production (Bertani et al., 2020). During the technological process of MBC, NWS plays a key role, for various reasons. First, NWS ensures rapid acidification of the curd, resulting in the modification of mineral equilibria and the modification of the curd into the stretching state, a distinctive and complex process that characterizes this product (Jana and Mandal, 2011; Pisano et al., 2016).

The use of NWS guides the fermentative process and the technology, which is the basis of the cheesemaking processes, and indeed, over the years, several authors have considered the study of MBC microbiota to be interesting, mainly investigated through culture-dependent studies (Parente et al., 1997; Morea et al., 1999; Coppola et al., 2001; Devirgiliis et al., 2008).

Going back over these studies, in recent years, there have been revolutionary changes in the study of food microbial ecology, starting from culture-dependent techniques, by means of culture-independent classical high-throughput sequencing (HTS) technologies. Nowadays, HTS has become a technique of choice for investigating the relative abundance at the taxon level of each microbial player that inhabits the ecosystem. This technique gives a complete and descriptive picture of the microbial community's complexity and composition (Widder et al., 2016).

HTS has proved to be a very useful and comprehensive technique for investigating different intermediate samples of MBC production, arising from two different producers, located in the provinces of Salerno and Caserta (Ercolini et al., 2012).

This study demonstrated that, despite the thought of this ecological complexity, only a few thermophilic LAB (*Streptococcus thermophilus, Lactobacillus delbrueckii*, and *Lactobacillus helveticus*) were the major players in curd fermentation; on the other hand, mesophilic microorganisms, such as *Lactococcus lactis* species, found in raw water buffalo milk became less abundant during manufacturing (Ercolini et al., 2012). Despite the small size of the MBC ecosystem, in terms of species able to guide the fermentative process, it has been extensively reported that the microbial NWS complexity and variability are strictly connected to the ecosystem, cheesemaking technology, and production area (Coppola et al., 1988, 2001; Bonizzi et al., 2007).

In this regard, it may be of interest to investigate several intermediate samples of MBC production, referring not only to different production areas but also to different production technologies, selecting a dairy that operates in a more traditional fashion and with smaller production capabilities, compared to a larger company that uses more modern equipment to produce larger volumes of product.

This study was performed to investigate the changes in the microbial structure of the MBC consortium throughout the cheesemaking process, considering variations between two dairy companies that varied in their production technologies and volumes, and in response to distinct technological steps, exploring the influences of selective and environmental pressures on the microbial community of MBC, as identified in this study.

## 2. Materials and methods

### 2.1. Selection of water buffalo mozzarella samples

A total of 22 samples (milk, NWS, cheese curd prior to stretching, brine samples, and mozzarella samples) were collected from two dairy companies, located in the Campania region, that were named M and C, respectively. The two dairy companies differ according to the technology in use. Dairy C is a smaller company that is run in a more traditional way, while dairy M is a larger producer with a modern operating dairy supply. Both companies must perform their cheesemaking according to the MBC PDO single document; however, some differences can be found in the MBC manufacturing, as shown in Table 1. For dairy M, the following samples were collected in duplicate from the same production day: pasteurized water buffalo milk (M), NWS, cheese curd prior to stretching (Cu), brine samples (Br), and mozzarella samples (Mo, 150 g each sample). For dairy C, the following samples were collected: water buffalo milk (tM) after thermization treatment (62°C for 15 s), NWS, Cu, two brine samples (BrNew and BrOld), and Mo (150 g each sample). Notably, the BrOld sample from dairy C has the peculiarity of having an old brine that has been in use for more than 30 years. All the samples were collected in duplicate, stored at 4°C until arrival at the lab, and frozen at −20°C until DNA extraction, except for brine samples, which were stored at a refrigerated temperature (4°C) until DNA extraction. Further details about the manufacturing conditions operated by each dairy are reported in Table 1.

**Table 1:** Details about the manufacturing dairies M and C, and of the technology applied for the MBC manufacturing.

**Dairy**	**M**	**C**
Size of the dairy	Large	Medium-small
Age of the milk at the time of cheesemaking (h)	Up to 50	Up to 24
Milk heat treatment	72°C for 20 s	62°C for 15 s
NWS acidity (SH/50 ml)	18	19
Renneting temperature °C	36	35.7
Rennet strength (IMCU)	220	235
Rennet% chymosin	Min. 95	75
Rennet amount (ml/100 L of milk)	18	8
Time of coagulation (from rennet addition to coagulum cutting; min)	25	65
Time of LAB fermentation (from NWS addition to cheese curd stretching; min)	240	320
Cheese curd temperature before stretching (°C)	25	27.5
Cheese curd pH before stretching	4.8	4.9
Cheese curd stretching conditions	Continuous with double diving arms equipment (capacity 500 kg/h)	Discontinuous, manual (20 kg for each batch)
Water temperature (°C)	92	93.5
Mode of addition of stretching water to curd	Continuous, with total recycling	New water for each curd batch
Mode of cheese shaping	Mechanical	Mechanical
Mode of mozzarella hardening	Dynamic	Static
Water temperature (°C)	18	22
Mode of brining	Dynamic	Static
Brine NaCl% (w/v)	4	8
Brine acidity (°SH 50 ml)	6.5	18
Brine temperature (°C)	16.5	19.5
Time of brining (150 g cheese ball; min)	60	25

### 2.2. DNA extraction and quantification

Total DNA extraction was performed using a DNeasy Blood and Tissue Kit (Qiagen, Hilden, Germany) with slight modifications. The following steps were performed to remove fat and milk impurities: for liquid samples (M, tM, NWS, and Br/BrO), 1 ml of sample was mixed with 2% (wt/vol) of sodium citrate solution in 10 ml of final volume and incubated in a water bath at 50°C for 30 min. For solid samples (Cu and Mo), 10 g of the solid matrix was homogenized in a sterile bag with 90 ml of 2% (wt/vol) of sodium citrate solution using a BagMixer (Interscience) for 2 min. Ten ml of the homogenized solid samples were collected and incubated at 50°C for 30 min in a water bath, as described for liquid samples. After incubation, the samples were centrifuged at 10,000 rpm for 10 min at 4°C, except for Br/BrO samples, which were centrifuged at 12,000 rpm for 30 min at 4°C. The supernatant and fat layers were removed, and the cells were resuspended in 10 ml of sodium citrate, repeating the washing step two times until all impurities were removed. The cell suspensions were centrifuged at 10,000 rpm for 10 min at 4°C. Subsequently, the manufacturer's protocol for DNA extraction from Gram-positive bacteria was followed, doubling the reagents' volumes. Briefly, the washed cell pellets were re-suspended in 360 μl of lysis buffer containing 25 mg/ml of lysozyme for 30 min at 37°C. The lysed cell suspension was treated with proteinase K for 30 min at 56°C. At the end of the spin-column protocol, the DNA was eluted with 50 μl of nuclease-free water, and the concentration and purity of the extracted nucleic acids were determined using a NanoDrop (NanoDrop™ 2000, Thermo Fisher Scientific, Waltham, Massachusetts, USA). DNA extraction from pasteurized buffalo milk samples from dairy M failed, whereas, for thermized buffalo milk from dairy C, pooling of the duplicate samples after DNA extraction was necessary to obtain sufficient material for the following analysis, resulting in a total of 19 samples with a concentration and purity of DNA suitable for 16S rRNA amplicon gene sequencing.

### 2.3. Bacterial composition analysis

Bacterial composition was evaluated in 19 samples from the two dairies. The V3–V4 region of the 16S rRNA gene was amplified and sequenced using previously described primers and PCR conditions (Takahashi et al., 2014). According to Takahashi et al. (2014), the amplification primers used are 341F (5′-CCTACGGGAGGCAGCAG-3′) and R806 (5′-GGACTACHVGGGTWTCTAAT-3′), and the PCR reaction conditions are as follows: initial denaturation at 98°C for 2 min, followed by 35 cycles of annealing beginning at 65°C and ending at 55°C for 15 s (−1°C/cycle), and a final extension at 68°C for 30 s. Library preparation and sequencing were carried out using BMR Genomics (Padova, Italy), on a MiSeq platform (Illumina Italy s.r.l., Milan, Italy), leading to 300 bp paired-ends fragments. The quality of the 16S rRNA amplicon raw reads was checked through FastQC (https://www.bioinformatics.babraham.ac.uk/projects/fastqc/). PRINSEQ was used for quality filtering (http://prinseq.sourceforge.net/), discarding raw reads shorter than 40 bp, and trimming bases with a Phred score below 20. Paired-end reads were joined through FLASH (Magoč and Salzberg, 2011). Merged sequences were then analyzed using QIIME 2 (v. 2022.11) software (Caporaso et al., 2010). The ASVs were picked using DADA2, and the most representative sequences of each cluster were selected in order to assign taxonomy using a Naïve Bayes classifier trained on the latest release of the Silva database (v. 138) (Quast et al., 2013; Bokulich et al., 2018; Robeson et al., 2021).

### 2.4. Statistical analysis

The QIIME2 file and phylogenetic tree were imported into R (version 4.2.2), and subsequent analyses were performed using packages *Phyloseq* (v. 1.42.0), Vegan (v. 2.6-4), and *DEseq2* (v 1.38.3). The *Phyloseq* package was used for data visualization. Prior to statistical analyses, two samples were discarded due to the extremely low number of sequences, while the raw milk sample was removed prior to phylogenetic and beta-diversity analyses.

The 27 ASVs with the highest cumulative abundance across all samples were selected to build a phylogenetic tree, and sequence assignment was verified using the BLAST algorithm against the rRNA/ITS database, setting a similarity threshold of 97%. Alpha-diversity significance was evaluated by analysis of variance (AOV) followed by *post-hoc* testing with the Kruskal–Wallis test, using the *-diversity* plugin from QIIME2. Differential ASV abundance was tested with *DESeq2* applying filtering of the sequences with a false discovery rate (FDR) cutoff of 0.01. Ordination was performed on compositional tables rarefied to the lowest abundance sequence count (25368 sequences), using the NMDS method and Bray–Curtis distance; the milk sample was not included in this analysis. To evaluate the significance of the clustering according to different dairies, permutational MANOVA analysis was carried out using the Adonis method, followed by the homogeneity of dispersion test (*betadisper*), to compare variability among the two classes, i.e., the different dairies (C or M).

## 3. Results

### 3.1. Microbial composition during MBC manufacturing steps

The 19 samples from the two dairies were submitted for amplicon sequencing. Two samples were excluded from this study due to the low quality of the obtained reads. A total of 680,000 reads were obtained after processing Illumina paired-end amplicons and quality filtering, with an average of 35,819 reads/sample; the number of sequences for each sample is shown in Supplementary Table 1. The species richness index (Chao1) and species diversity indices (Simpson and Shannon) were calculated for all samples after the rarefaction step and are fully shown in Supplementary Table 2.

**Figure 1:**
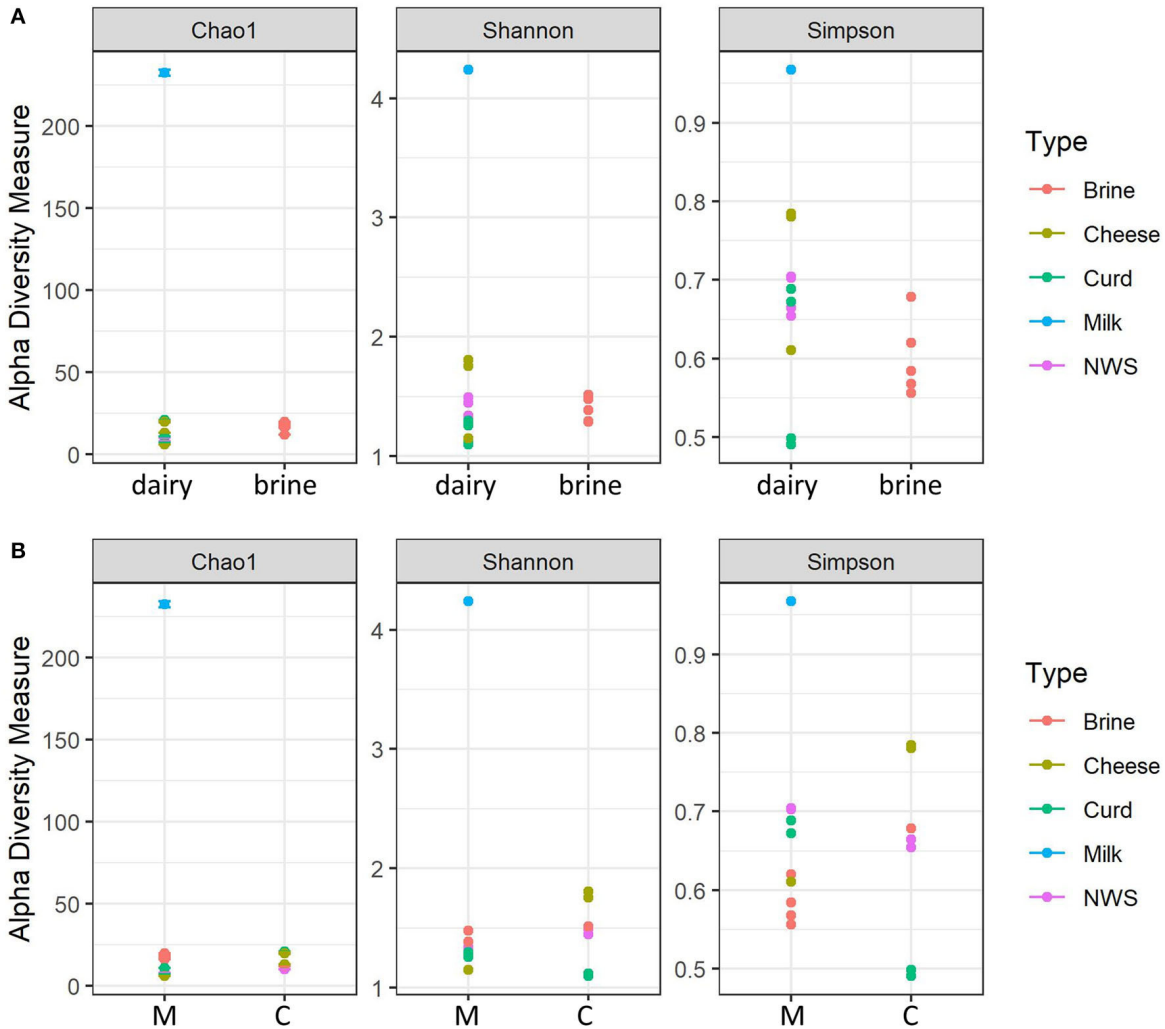
Alpha-diversity indices calculated for water buffalo mozzarella samples. **(A)** Alpha-diversity according to the nature of the samples (dairy = Cheese, curd, milk, NWS). **(B)** Alpha-diversity according to dairy production plant: M, dairy M; C, dairy C.

Figure 1 shows the distribution of the alpha-diversity metrics, which were grouped according to their sample type (dairy or brine, Figure 1A), or dairy company (C or M, Figure 1B). Brine samples show a comparable richness in terms of species compared to dairy samples, except for the milk sample, which shows the highest diversity for all indices (Figure 1A). Considering evenness metrics, dairy samples tend to differ from brine samples, but there are some distinctions according to the different manufacturing stages. This is even more evident for curd samples, where few species develop and take over the fermentation process (Figure 1A). Notably, in both displays, the thermized milk sample used from company C for cheesemaking shows the highest number of observed species, as well as the highest diversity (Shannon) and low dominance (Simpson) indicators. Cheesemaking technology greatly affects alpha-diversity indices, and Cu and Mo samples manufactured from heat-treated milk undergo a decrease in species richness and diversity (Figure 1B). In general, species richness of samples from dairy M, in which milk has been pasteurized, was lower than that observed for dairy C, while similar trends were observed for the other indices (Figure 1B).

The identified sequences were assigned to 30 different phyla, among which Firmicutes represented the majority *phylum* in all samples, ranging between 99.0 and 100% of the population, except for milk samples, in which its abundance is 40.2%. The microbiota of milk samples is characterized by the presence of other *phyla*, such as Proteobacteria (34.1%), Actinobacteriota (21.5%), and Bacteroidota (3.6%), as well as other minority phyla below 1% abundance. Among Firmicutes, Bacilli are the most represented class (ranging between 98.7 and 100%) in all samples except milk, representing 35.1% of the bacterial composition, followed by Gammaproteobacteria (33.8%), Actinobacteria (21.5%), and Clostridia (5%).

**Figure 2:**
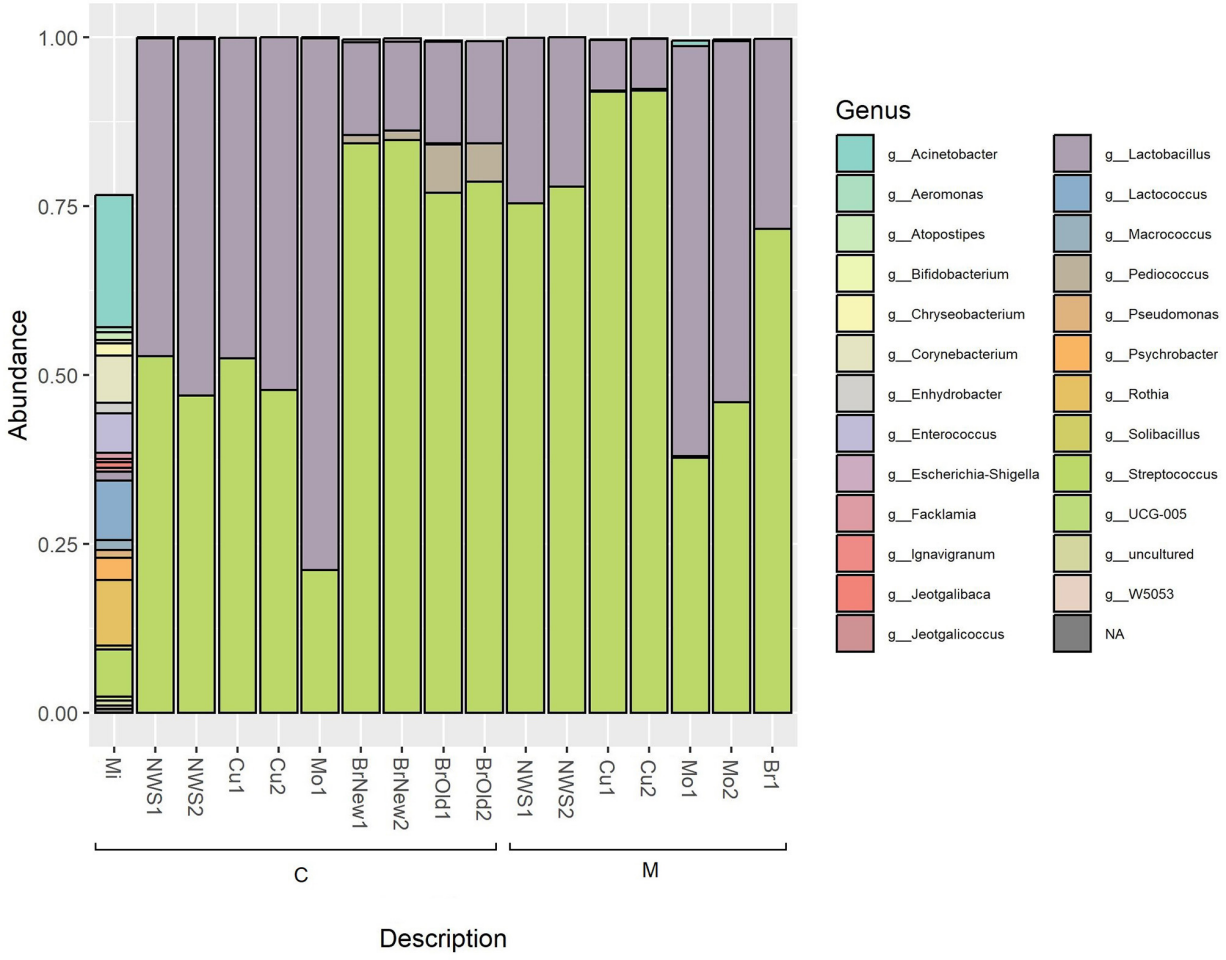
Relative abundance of taxa based on 16S rRNA amplicon sequencing; only bacterial genera with an abundance above 0.1% are shown.

The total identified ASVs were grouped according to their taxonomic assignment, and only the ASVs whose abundance was above 0.1% in at least one sample were retained (Table 2). This abundance filtering allowed a total of 26 taxa identified at the genus level to be retained, as shown in Figure 2. The milk sample used for cheesemaking in dairy C maintains a relevant diversity in its composition and is dominated by *Acinetobacter* (22.0%), *Rothia* (9.7%), *Lactococcus*, and *Corynebacterium* at the same extent (8.9%), as well as *Streptococcus* (7.4%) and *Enterococcus* (6.5%). The composition of the NWS is different for the two dairies: NWS from dairy C shows a similar abundance of the genera *Streptococcus* (49.9 ± 4.1%) and *Lactobacillus* (49.9 ± 4%), while the NWS from dairy M is dominated by *Streptococcus* (76.7 ± 1.8%), followed by *Lactobacillus* (23.3 ± 1.8%). The composition of curd samples reflects this slight difference in species proportion, and after the curd acidification step, in which the pH decreases to ~4.8, samples from dairy M show a dominance of genus *Streptococcus* (92.1 ± 0%) over *Lactobacillus* (7.5 ± 0.1%), while samples from dairy C show a balanced abundance of *Streptococcus* (50.1 ± 3.3%) and *Lactobacillus* (49.8 ± 3.3%). After the curdling step, the curd is melted with boiling water, stretched, and then molded into its final shape, hardened in flowing tap water, and then submerged in brine for 25–60 min (see Table 1).

**Table 2:** Relative abundance of the ASVs shows an abundance of at least 0.5% in at least one sample.

**Genus**	**C**										**M**						
	**tM**	**NWS1**	**NWS2**	**BrOld1**	**BrOld2**	**BrNew1**	**BrNew2**	**Cu1**	**Cu2**	**Mo1**	**NWS1**	**NWS2**	**Br1**	**Cu1**	**Cu2**	**Mo1**	**Mo2**
*Lactococcus*	8.8	0	0	0.21	0	0	0	0	0	0	0	0	0	0.13	0.23	0.22	0
*Streptococcus*	6.95	52.81	46.98	76.98	78.65	84.28	84.79	52.47	47.8	21.14	75.39	77.92	71.64	91.96	92.12	37.73	45.98
*Lactobacillus*	1.29	47.06	52.77	15.01	15.14	13.71	13.13	47.43	52.18	78.73	24.54	22.08	28.13	7.53	7.43	60.73	53.44
*Pediococcus*	0	0	0	7.13	5.67	1.29	1.41	0	0	0	0	0	0	0	0	0	0
*W5053*	0.52	0	0	0	0	0	0	0	0	0	0	0	0	0	0	0	0
*UCG-005*	0.56	0	0	0	0	0	0	0	0	0	0	0	0	0	0	0	0
*Pseudomonas*	1.16	0	0	0	0	0	0	0	0	0	0	0	0	0	0	0	0
*Enhydrobacter*	1.58	0	0	0	0	0	0	0	0	0	0	0	0	0	0	0	0
*Psychrobacter*	3.32	0	0	0	0	0	0	0	0	0	0	0	0	0	0	0	0
*Acinetobacter*	19.57	0	0	0	0	0	0	0	0	0	0	0	0	0	0	0.88	0.22
*Aeromonas*	0.71	0	0	0	0	0	0	0	0	0	0	0	0	0	0	0	0
*Enterobacteriaceae*	0.54	0	0	0	0	0	0	0	0	0	0	0	0	0	0	0	0
*Escherichia-Shigella*	0	0.13	0.25	0.22	0	0.39	0.56	0.05	0	0.12	0	0	0	0	0	0	0
*Chryseobacterium*	1.78	0	0	0	0	0	0	0	0	0	0	0	0	0	0.05	0	0
*Corynebacterium*	6.97	0	0	0	0	0	0	0	0	0	0	0	0	0.03	0	0	0
*Bifidobacterium*	0.54	0	0	0	0	0	0	0	0	0	0	0	0	0	0	0	0
*Rothia*	9.73	0	0	0	0	0	0	0	0	0	0	0	0	0	0	0	0
*Jeotgalicoccus*	0.55	0	0	0	0	0	0	0	0	0	0	0	0	0	0	0	0
*Macrococcus*	1.49	0	0	0	0	0	0	0	0	0	0	0	0	0	0	0	0
*Solibacillus*	0.58	0	0	0	0	0	0	0	0	0	0	0	0	0	0	0	0
*Jeotgalibaca*	0.85	0	0	0	0	0	0	0	0	0	0	0	0	0	0	0	0
*Enterococcus*	5.88	0	0	0	0	0	0	0	0	0	0	0	0	0	0	0	0
*Atopostipes*	1.15	0	0	0	0	0	0	0	0	0	0	0	0	0	0	0	0
*Facklamia*	0.86	0	0	0	0	0	0	0	0	0	0	0	0	0	0	0	0
*uncultured*	0.76	0	0	0	0	0	0	0	0	0	0	0	0	0	0	0	0
*Ignavigranum*	0.52	0	0	0	0	0	0	0	0	0	0	0	0	0	0	0	0
Total	76.65	100	100	99.55	99.46	99.66	99.88	99.95	99.98	100	99.93	100	99.77	99.65	99.83	99.56	99.64

Taxa were collapsed according to the identified genera. Data are reported as relative abundance %.

After this step, the relative abundance of the genus *Lactobacillus* in mozzarella cheese from both dairies increases to 78.7% in mozzarella from dairy C and 57.1 ± 5.2% in mozzarella from dairy M. At the same time, in this manufacturing step, the relative abundance of the genus *Streptococcus* decreases, reaching 21.1% in mozzarella from dairy C and 41.9% in dairy M. These two genera account for more than 90% of the microbial composition of these samples.

The brine used in both dairies is dominated by the genus *Streptococcus*, with higher values for dairy C (84.5 ± 0.4%) compared to dairy M (71.6%), followed by the *Lactobacillus* genus, which has a larger abundance in dairy M (28.1%) compared to dairy C (13.4 ± 0.4%).

Interestingly, dairy C has been using another kind of brine for more than 30 years with periodic treatments. This brine was not used in the Mo sample treatment in this study, but it was sampled as a source of microbiological diversity. Comparing the two brines from this company, we can observe that the older one (BrO) is characterized by a higher abundance of genus *Pediococcus* (6.4 ± 1.0%) compared to the newer one (BrNew, 1.3 ± 0.1%). The already encountered genus *Streptococcus* (77.9 ± 1.1% for the BrOld and 84.5 ± 0.4% for the BrNew) and *Lactobacillus* (15.2 ± 0.1% for the BrOld, and 13.6 ± 0.6% for the BrNew) are present as well and are prevalent in brine from dairy M (71.6% *Streptococcus*, 28.1% *Lactobacillus*), where genus *Pediococcus* was not identified.

### 3.2. Phylogenetic relationships of the samples' microbiome

**Figure 3:**
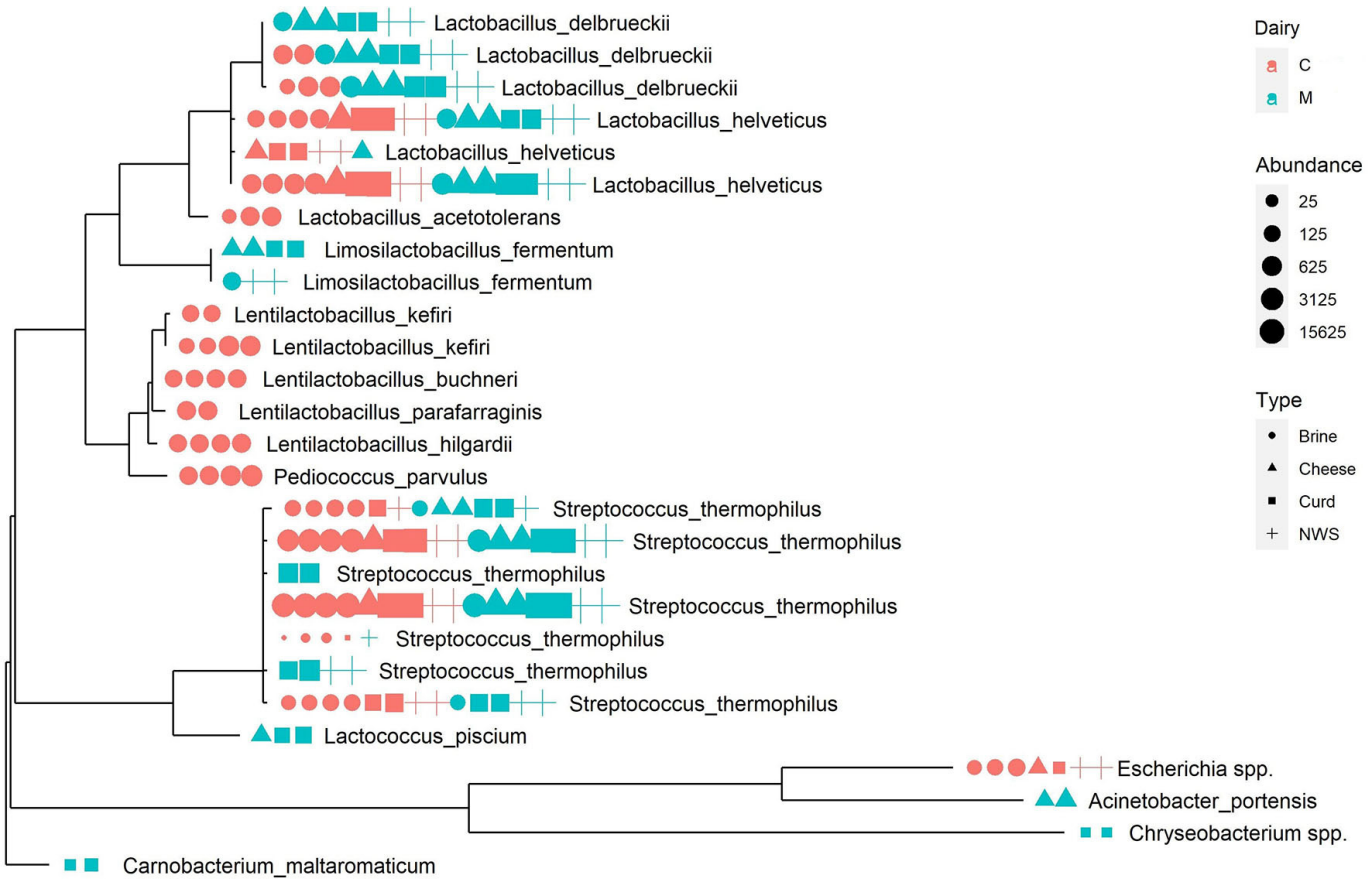
Phylogenetic tree representing the most abundant ASVs in the dataset. Next to each tree tip, a graphic representation summarizes the abundance of the sequence in each sample, colored according to production dairy, shaped according to the sample type, and sized according to the absolute abundance.

A phylogenetic tree was built to describe the taxonomic relationships occurring among ASVs (Figure 3). As already observed from the compositional analysis of relative abundance data, samples are dominated by the genera *Streptococcus*, mainly represented by the species *S. thermophilus* and *Streptococcus salivarius*, and by genus *Lactobacillus*, particularly the species *L. helveticus* and *L. delbrueckii*.

The ASVs from the *Lactobacillus* genus are widespread among all samples but, interestingly, some specific ASVs are related to the provenance dairy, as is the case of one *L. delbrueckii* ASV represented in all samples from dairy M, at a relevant abundance, but absent in the microbiome of dairy C (Figure 3).

Similarly, the genus *Streptococcus*, which is dominant in all samples (Table 2), shows some interesting patterns of dairy-specific ASVs, as in the case of *S. thermophilus* and *S. salivarius* identified exclusively in the curd and NWS of dairy M (Figure 3). Interestingly, another *S. thermophilus* ASV is present only in brine, NWS, and curd from both dairies, but absent in mozzarella cheese samples (Figure 3), suggesting that the related strain could be more adapted to the early stages of cheese manufacturing. Phylogenetic analysis helps to define more clearly the species assignment inside the genus *Streptococcus*, showing the close relatedness of the corresponding ASVs, which suggests they belong to the *S. thermophilus* species.

Finally, some ASVs from the genus *Lentilactobacillus* and one from *Pediococcus* form a distinct phylogenetic cluster that is related to brine samples from dairy C (Figure 3). It is interesting to remark that these species are strongly associated with the brine environment and are never identified in the remaining dairy C samples.

Low-abundance species, such as *Lactococcus piscium, Acinetobacter portensis, Chryseobacterium* spp., and *Carnobacterium maltaromaticum*, are associated with cheese and curd from dairy M, as is the case of *Escherichia* spp. related to dairy C samples. These low-abundance species are phylogenetically more distant from the LAB species making the most of the samples' microbiomes and forming separated clusters on the phylogenetic tree (Figure 3).

**Figure 4:**
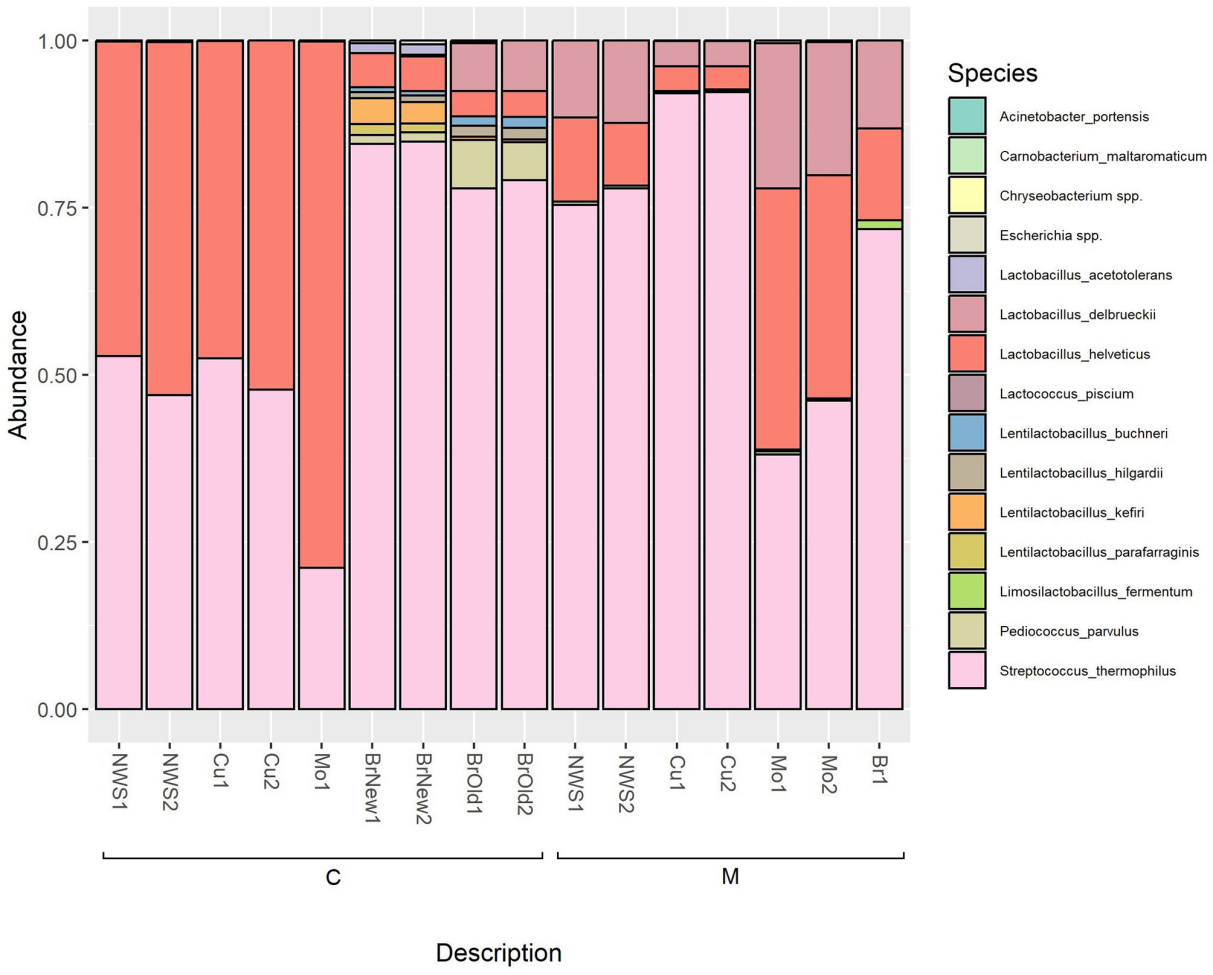
Relative abundance of ASVs at the species level based on 16S rRNA sequencing; only bacterial genera with an abundance above 0.5% are shown.

As shown in Figure 4, and reported in Supplementary Table 3, the microbiome of curd and NWS of samples produced by dairy C are dominated by the species *L. helveticus*, which increases further after the “filatura” process, representing over 75% of the species composition of the obtained Mo sample, while the relative abundance of the species *S. thermophilus* decreases. Interestingly, in both the brine samples from this dairy (BrNew and BrOld), there is a dominance of *S. thermophilus*, while *L. helveticus* is a minority. In both brine solutions, especially BrOld samples, *L. delbrueckii* was detected, but not in any other samples from the same dairy.

Similarly, in the samples collected in dairy M, we observe an initial dominance of the species *S. thermophilus* in NWS and curd, while the *Lactobacillus* species *L. helveticus* and *L. delbrueckii* represent <25% of the microbiome of NWS samples and <10% of the curd microbiome after the stretching step. However, the resulting mozzarella shows a predominance of the *Lactobacillus* species, consisting of over 50% of the cheese microbiome.

### 3.3. Structure of the microbiota

**Figure 5:**
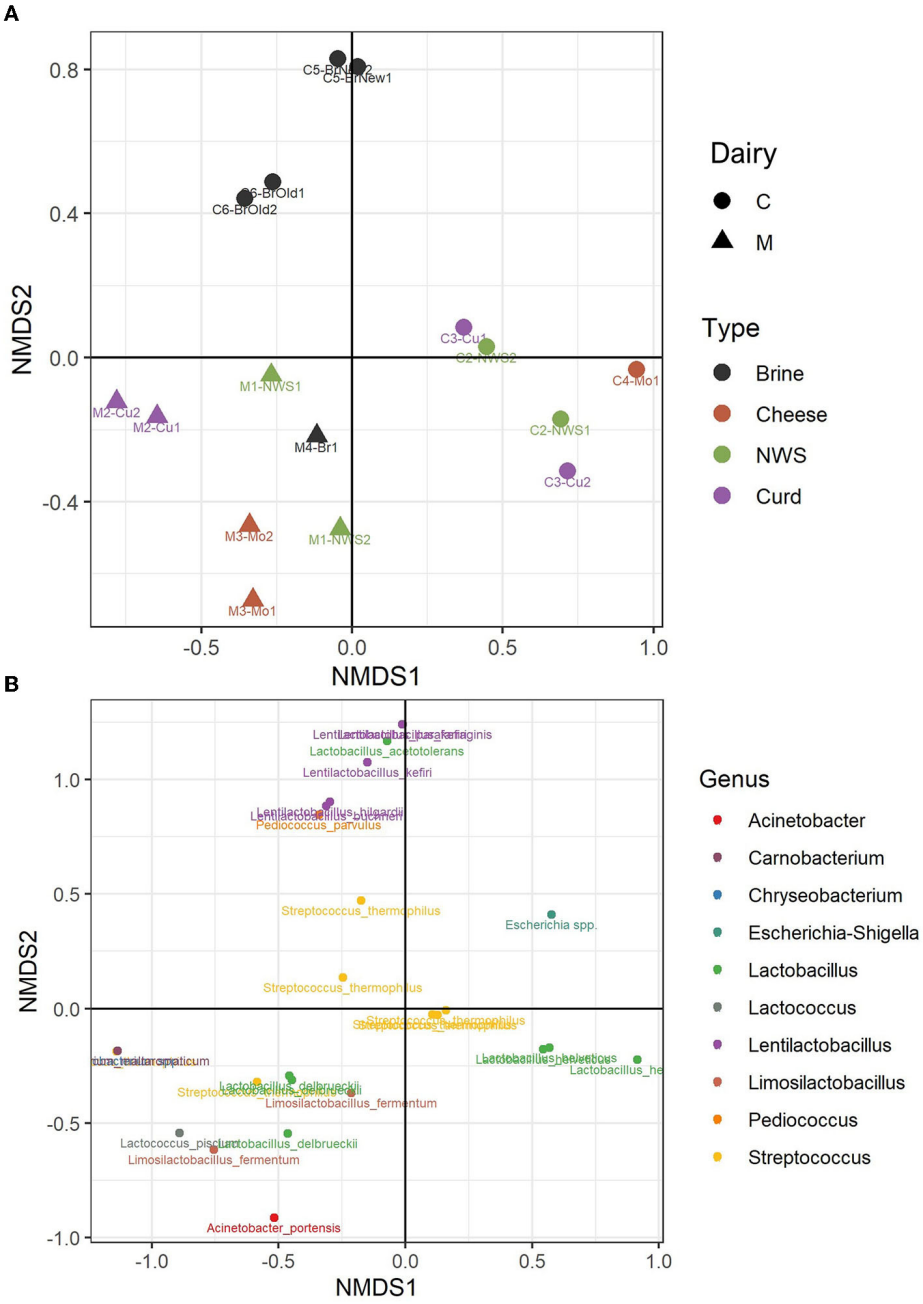
Non-metric dimensional scaling (NMDS) of water buffalo mozzarella samples according to their microbiota. **(A)** Clustering of samples from dairy C (circles) and dairy M (triangles); samples are colored according to the sample type. **(B)** Distribution of bacterial ASVs along the NMDS axes; taxa are colored according to the assigned genus.

A beta diversity analysis was performed using the NMDS method and Bray–Curtis distance method. Figure 5A shows how the two dimensions clearly distinguish dairy products from dairies C and M, in the left and right lower quadrants of the sample plots (Figure 5A). However, the samples produced by the two dairies, M and C, do not show a strong compositional dissimilarity, as demonstrated by similar group dispersion and confirmed by no statistically significant differences (ADONIS, *p* > 0.05, Supplementary Figure 1). However, some differences can be observed among samples. Indeed, the samples of dairy C show a clear distinction among the composition of the brine samples, which cluster together in the upper half of the plot, driven by the presence of the genera *Pediococcus* and *Lentilactobacillus*, which are absent from the curd, NWS, and mozzarella samples from the same dairy (Figure 5B). Clustering of samples from dairy M is driven by the species *L. delbrueckii*.

Samples from dairy M, located in the lower part of the NMDS graph (Figure 5A), show a more divergent composition in the ecology of NWS, curd, and cheese samples. According to the taxa plot (Figure 5B), these differences are driven by the presence of minority genera *Lactococcus, Limosilactobacillus*, or *Acinetobacter*, which are present at low abundance, and by the species *L. delbrueckii* (Supplementary Table 3). On the other hand, samples from dairy C show more similarity in the composition of curd, NWS, and mozzarella samples (Figure 5A), with more ASVs belonging to the species *L. helveticus*. The species *S. thermophilus*, which dominates the microbiota of most of the samples from both dairies, is in the central area of the NMDS graph.

## 4. Discussion

In this study, the microbial composition was evaluated at different stages of MBC PDO cheesemaking, including milk, NWS, curd, and cheese samples. Cheese brine was investigated in this study as it is a key point of MBC PDO cheese, different from many other mozzarella cheeses in which salting takes place during the stretching operation (Gobbetti et al., 2018). Therefore, although MBC is a fresh cheese, brining can be regarded as a potential reservoir of microbial diversity because brine microbiota contaminates the MBC surface. Various authors have investigated the composition of different types of mozzarella and pasta filata cheeses using the HTS approach to describe the microbiota of these dairy products. The composition of NWS used for the production of water buffalo mozzarella has been investigated by De Filippis et al. (2014), confirming that the dominating species are *S. thermophilus*, followed by *L. delbrueckii* and *L. helveticus*. In agreement with this observation, the analyzed samples show a prevalence of *Streptococcus* and *Lactobacillus* spp., and the ratio among these two taxa is slightly different between the two dairies, possibly an interesting aspect of dairy-specific variations to be investigated more in-depth. The presence of *S. thermophilus* in NWS used for manufacturing mozzarella is well-documented in cheeses produced with cow's or buffalo's milk (De Filippis et al., 2014; Guidone et al., 2016; Marino et al., 2019). Previous studies have shown that a certain degree of diversity exists within dairy isolates of this species originating from mozzarella cheese (Silva et al., 2019), and this diversity can be observed as well in terms of amplicon-based sequence diversity (De Filippis et al., 2014). In this study, various ASVs identified as *S. thermophilus*, are present in all samples, as well as among the most abundant taxa, including raw milk; and no signature ASVs were identified that could be related to either one of the dairy companies. The higher relative abundance of the species *L. helveticus* in samples from dairy C could be explained by some key manufacturing steps. Looking at the data reported in Table 1, it appears that in dairy C a lower amount of rennet is added prior to the curdling process compared to dairy M. Therefore, the coagulation and fermentation times are prolonged for dairy C. These conditions will favor slower coagulation, which could be related to the higher abundance of lactobacilli in dairy C curd and the higher concentrations found in the resultant MBC sample.

Buffalo's thermized milk (dairy C) showed the greatest bacterial diversity among samples, as highlighted by the alpha-diversity results, while pasteurized milk (dairy M) did not allow for sufficient DNA amplification. This finding adds to the general *consensus* in the literature that raw milk has a complex microbiota (Quigley et al., 2013; Parente et al., 2020; Bettera et al., 2023). A minimal number of studies have investigated the composition of raw buffalo's milk by means of the HTS approach, revealing the dominance of species belonging to the genera *Enterococcus, Streptococcus, Lactococcus, Acinetobacter*, and *Psychrobacter* (Ercolini et al., 2012; Catozzi et al., 2017). This study, probably for the first time, shows that mild conditions of thermization of buffalo's milk maintain a higher degree of biodiversity. Particularly, the psychrotrophic genera *Acinetobacter* and *Psychrobacter*, retrieved in this study as well, have been linked to refrigerated storage or bulk and transport tank contamination in studies performed on cow's milk (Falardeau et al., 2019; Zhang et al., 2019). The increase in the relative abundance of thermophilic LABs within the time of curd fermentation and higher heat sensitivity of the previous genera might explain their absence in curd and MBC samples.

After the addition of NWS, the bacterial community in the cheese curd is dominated by the species *S. thermophilus*, followed by the species *L. helveticus* and *L. delbrueckii*. These lactobacilli are frequently found in cheese curds of many varieties, and the dairy-related prevalence of the species *L. helveticus* in curd from water buffalo mozzarella has already been reported by Ercolini et al. (2012). After molding into the final shape, the mozzarella curds are salted by brining. The microbiota of the brine used for manufacturing water buffalo mozzarella was not described earlier using HTS technologies, but previous studies indicate that the findings of the genera *Lactobacillus* and *Streptococcus* are common in cheese brines, as well as the minority genera *Pediococcus* (Marino et al., 2017; Haastrup et al., 2018). Among the genus *Lactobacillus*, various species were detected in brine samples, such as *L. helveticus* and *L. delbrueckii*, that have already been identified in Danish cheese brine (Haastrup et al., 2018), as well as the previously undetected *Lactobacillus kefiri, Lactobacillus fermentum, Pediococcus parvulus, Lactobacillus buchneri, Lactobacillus parafarraginis*, and *Lactobacillus hilgardii*. It is noteworthy that the brines prepared by the dairies are different in terms of NaCl%, acidity, and temperature (Table 1), and these differences could explain the different microbial compositions of brines. Interestingly, the species that were detected exclusively in a brine of dairy C are not present in the resulting mozzarella, probably because of a low degree of contamination of the cheese surface. This might suggest that these species might be well-adapted to the brine environment, but do not determine variations in the composition of the resulting mozzarella cheeses, or possibly they could be able to develop in the MBC microbiome within the time of the shelf-life, which was not further investigated in this study. The final mozzarella samples from both dairies are characterized by an inversion in the microbiota dominance of the *Lactobacillus* genus over *Streptococcus*, possibly due to the relief of the thermal stress after the curd stretching step (Mucchetti and Neviani, 2006). At the same time, the species *L. helveticus* and *L. delbrueckii* could have a higher tolerance to the combined exposure to thermal stress and pH reduction, considering that the pH drops below 4.9 during the curd maturation process. Particularly, the species *L. helveticus* is more abundant in mozzarella produced from dairy C which showed a higher percentage of this species in the NWS used for manufacturing, while the species *L. delbrueckii* is more abundant in mozzarella from dairy M, in agreement with a higher relative abundance in the NWS. The reduction of the genus *Streptococcus* after the curd formation step has been documented by Ercolini et al. (2012), and an increased abundance of the genus *Lactobacillus* is observed in water buffalo mozzarella samples. At the same time, the curd stretching step causes a decrease in the microbial load reached during the curd maturation step because of the combined effect of temperature and pH (Mucchetti and Neviani, 2006). Following this, the species *L. helveticus*, which is thermoresistant and aciduric, dominates the microbiome of MBC, followed by the thermoresistant species *L. delbrueckii*, if present, as already shown in other dairy ecosystems (Bertani et al., 2020). A comparison with cow high-moisture mozzarella samples would be interesting as curd is stretched at a higher pH (~5.2) and low temperature (usually <60°C). In that case, *Streptococcus* is by far the dominant genus (Marino et al., 2019) but comparison is impossible because lactobacilli are not used as a starter.

Interestingly, in this study, no ASVs were assigned to the species *Lactobacillus fermentum* which is reported to be present in NWS, curd, and water buffalo mozzarella in a dairy-dependent fashion (De Filippis et al., 2014).

Cheeses, particularly artisanal cheeses, are rarely the result of the activities of an individual but that of a group of microorganisms. Most food fermentation processes depend on mixtures of microorganisms (species and biotypes), which act in concert to produce the desired product characteristics. Cheeses are often characterized by the presence of a complex microbiota and have to be discussed taking into account the scenario of microbial communities. In this perspective, MBC has a dynamic ecosystem in which players are subjected to continuous temporal environmental/technological stimuli. In this scenario, it is of great interest that the MBC cheesemaking process, despite the initial presence of a large and varied microbiota, is mainly in the hands of a small microbiome, mainly composed of *Streptococcus* and *Lactobacillus* spp, supporting the conclusion that the composition of the NWS represents the key factor that drives the composition of the final MBC products. Small differences in the application of the cheesemaking technology, supported by each cheesemaker and falling within the PDO manufacturing specifications, are more likely to explain dairy-to-dairy variation and characteristics.

## Data availability statement

The NGS data presented in the study are deposited in the Sequence Read Archive (SRA), BioProject ID: PRJNA950113.

## Author contributions

AL, VB, and CL contributed to the conception and design of the study. AL and GB performed bioinformatics analyses and wrote the first draft of the manuscript. AL performed statistical analyses. GB and MM performed laboratory experiments. GM, EN, VB, and CL provided technical insight and critically revised the manuscript. All authors have contributed to the manuscript revision, have read, and approved the submitted version.
