# Supplementary material for: The microbiota of Mozzarella di Bufala Campana PDO cheese: a study across the manufacturing process
Source: Front Microbiol. 2023 Aug 15;14:1196879. doi: 10.3389/fmicb.2023.1196879 (PMC10462780; doi:10.3389/fmicb.2023.1196879)
Supplement: Supplementary file 1 [file Data_Sheet_1.docx]

Supplementary Material

The microbiome of Mozzarella di Bufala Campana PDO cheese: a study across the manufacturing process

Alessia Levante^1*^, Gaia Bertani^1^, Martina Marrella^1^, Germano Mucchetti^1^, Valentina Bernini^1^, Camilla Lazzi^1^, Erasmo Neviani^1^.

*** Correspondence:** Alessia Levante, alessia.levante@unipr.it

# Supplementary Figures and Tables

**Supplementary table 1:** Number of sequences retrieved in each sample after quality filtering.

| **Dairy** | **Sample Name** | **Type** | **Number of reads** |
| --- | --- | --- | --- |
| **C** | NWS1 | NWS | 35423 |
| **C** | NWS2 | NWS | 32048 |
| **C** | BrOld1 | Brine | 37534 |
| **C** | BrOld2 | Brine | 27475 |
| **C** | BrNew1 | Brine | 41617 |
| **C** | BrNew2 | Brine | 47933 |
| **C** | Cu1 | Curd | 46384 |
| **C** | Cu2 | Curd | 46590 |
| **C** | Mo1 | Cheese | 50508 |
| **C** | Mo2 | Cheese | 0 |
| **C** | tM | Milk | 41966 |
| **M** | NWS1 | NWS | 46126 |
| **M** | NWS2 | NWS | 39680 |
| **M** | Br1 | Brine | 31889 |
| **M** | Br2 | Brine | 0 |
| **M** | Cu1 | Curd | 51841 |
| **M** | Cu2 | Curd | 46478 |
| **M** | Mo1 | Cheese | 31617 |
| **M** | Mo2 | Cheese | 25459 |
|  |  | **Avg**. | 35819 |

**Supplementary table 2:** Diversity indices for 16S rRNA amplicons from MBC samples. Data are obtained after multiple (10) rarefaction of samples at 35,819 reads. Data of chao1 indicator are reported as mean and standard error (se.chao1)

| **Dairy** | **Sample** | **Replicate** | **Chao1** | **se.chao1** | **Shannon** | **Simpson** |
| --- | --- | --- | --- | --- | --- | --- |
| **Dairy C** | Milk | tM | 232.25 | 1.732623 | 4.236996 | 0.967055 |
|  | NWS | NWS1 | 7 | 0 | 1.32639 | 0.702897 |
|  |  | NWS2 | 8 | 0 | 1.340505 | 0.704252 |
|  | Brine (old) | BrOld1 | 20 | 0 | 1.477128 | 0.619854 |
|  |  | BrOld2 | 16 | 0 | 1.384504 | 0.583973 |
|  | Brine (new) | BrNew1 | 18 | 0.242956 | 1.290039 | 0.556391 |
|  |  | BrNew2 | 17 | 0 | 1.29511 | 0.567957 |
|  | Curd | Cu1 | 11 | 0 | 1.297936 | 0.68849 |
|  |  | Cu2 | 7 | 0 | 1.255568 | 0.672388 |
|  | Cheese | Mo1 | 6 | 0 | 1.148095 | 0.610886 |
| **Dairy M** | NWS | NWS1 | 13 | 0 | 1.491137 | 0.66432 |
|  |  | NWS2 | 10 | 0 | 1.447266 | 0.654368 |
|  | Brine | Br1 | 12 | 0 | 1.510968 | 0.678477 |
|  | Curd | Cu1 | 20 | 0.48734 | 1.119284 | 0.498982 |
|  |  | Cu2 | 21 | 0 | 1.097014 | 0.490812 |
|  | Cheese | Mo1 | 20 | 0.48734 | 1.808141 | 0.784737 |
|  |  | Mo2 | 13 | 0 | 1.752253 | 0.780876 |

**Supplementary table 3:** Relative abundance of the ASVs after species identification. Only ASVs whose abundance was higher than 0.1% in at least one sample are reported

|  | **Dairy C** | | | | | | | | | | | | | | **Dairy M** | | | | | | | | | |  |
| --- | --- | --- | --- | --- | --- | --- | --- | --- | --- | --- | --- | --- | --- | --- | --- | --- | --- | --- | --- | --- | --- | --- | --- | --- | --- |
|  | **NWS** | | **Brine (old)** | | | | **Brine (new)** | | **Curd** | | | **Cheese** | | | **NWS** | | **Brine** | | **Curd** | | | | **Cheese** | |  |
| **Species** | **NWS1** | **NWS2** | | **BrOld1** | **BrOld2** | **BrNew1** | | **BrNew2** | | **Cu1** | **Cu2** | | **Mo1** | **NWS1** | | **NWS2** | | **Br1** | | **Cu1** | **Cu2** | **Mo1** | | **Mo2** | |
| *Lactococcus piscium* | 0.00 | 0.00 | | 0.00 | 0.00 | 0.00 | | 0.00 | | 0.00 | 0.00 | | 0.00 | 0.00 | | 0.00 | | 0.00 | | 0.13 | 0.23 | 0.23 | | 0.00 | |
| *Streptococcus thermophilus* | 52.81 | 46.98 | | 77.89 | 79.08 | 84.56 | | 84.89 | | 52.51 | 47.81 | | 21.14 | 75.45 | | 77.92 | | 71.80 | | 92.09 | 92.26 | 38.08 | | 46.14 | |
| *Limosilactobacillus fermentum* | 0.00 | 0.00 | | 0.00 | 0.00 | 0.00 | | 0.00 | | 0.00 | 0.00 | | 0.00 | 0.45 | | 0.37 | | 1.28 | | 0.22 | 0.23 | 0.49 | | 0.35 | |
| *Lactobacillus acetotolerans* | 0.00 | 0.00 | | 0.16 | 0.00 | 1.53 | | 1.59 | | 0.00 | 0.00 | | 0.00 | 0.00 | | 0.00 | | 0.00 | | 0.00 | 0.00 | 0.00 | | 0.00 | |
| *Lactobacillus helveticus* | 47.06 | 52.77 | | 3.73 | 3.85 | 5.05 | | 5.19 | | 47.45 | 52.19 | | 78.73 | 12.64 | | 9.38 | | 13.79 | | 3.69 | 3.42 | 39.13 | | 33.33 | |
| *Lactobacillus delbrueckii* | 0.00 | 0.00 | | 7.20 | 7.54 | 0.00 | | 0.22 | | 0.00 | 0.00 | | 0.00 | 11.47 | | 12.33 | | 13.13 | | 3.78 | 3.80 | 21.66 | | 19.95 | |
| *Lentilactobacillus hilgardii* | 0.00 | 0.00 | | 1.68 | 1.75 | 0.92 | | 0.98 | | 0.00 | 0.00 | | 0.00 | 0.00 | | 0.00 | | 0.00 | | 0.00 | 0.00 | 0.00 | | 0.00 | |
| *Lentilactobacillus parafarraginis* | 0.00 | 0.00 | | 0.00 | 0.00 | 1.63 | | 1.30 | | 0.00 | 0.00 | | 0.00 | 0.00 | | 0.00 | | 0.00 | | 0.00 | 0.00 | 0.00 | | 0.00 | |
| *Lentilactobacillus buchneri* | 0.00 | 0.00 | | 1.42 | 1.70 | 0.78 | | 0.63 | | 0.00 | 0.00 | | 0.00 | 0.00 | | 0.00 | | 0.00 | | 0.00 | 0.00 | 0.00 | | 0.00 | |
| *Lentilactobacillus kefiri* | 0.00 | 0.00 | | 0.48 | 0.39 | 3.85 | | 3.23 | | 0.00 | 0.00 | | 0.00 | 0.00 | | 0.00 | | 0.00 | | 0.00 | 0.00 | 0.00 | | 0.00 | |
| *Pediococcus parvulus* | 0.00 | 0.00 | | 7.22 | 5.70 | 1.29 | | 1.41 | | 0.00 | 0.00 | | 0.00 | 0.00 | | 0.00 | | 0.00 | | 0.00 | 0.00 | 0.00 | | 0.00 | |
| *Acinetobacter portensis* | 0.00 | 0.00 | | 0.00 | 0.00 | 0.00 | | 0.00 | | 0.00 | 0.00 | | 0.00 | 0.00 | | 0.00 | | 0.00 | | 0.00 | 0.00 | 0.41 | | 0.22 | |
| *Escherichia spp.* | 0.13 | 0.25 | | 0.22 | 0.00 | 0.39 | | 0.56 | | 0.05 | 0.00 | | 0.12 | 0.00 | | 0.00 | | 0.00 | | 0.00 | 0.00 | 0.00 | | 0.00 | |
| *Chryseobacterium spp.* | 0.00 | 0.00 | | 0.00 | 0.00 | 0.00 | | 0.00 | | 0.00 | 0.00 | | 0.00 | 0.00 | | 0.00 | | 0.00 | | 0.02 | 0.03 | 0.00 | | 0.00 | |
| *Carnobacterium maltaromaticum* | 0.00 | 0.00 | | 0.00 | 0.00 | 0.00 | | 0.00 | | 0.00 | 0.00 | | 0.00 | 0.00 | | 0.00 | | 0.00 | | 0.06 | 0.04 | 0.00 | | 0.00 | |

## Supplementary Figures

**
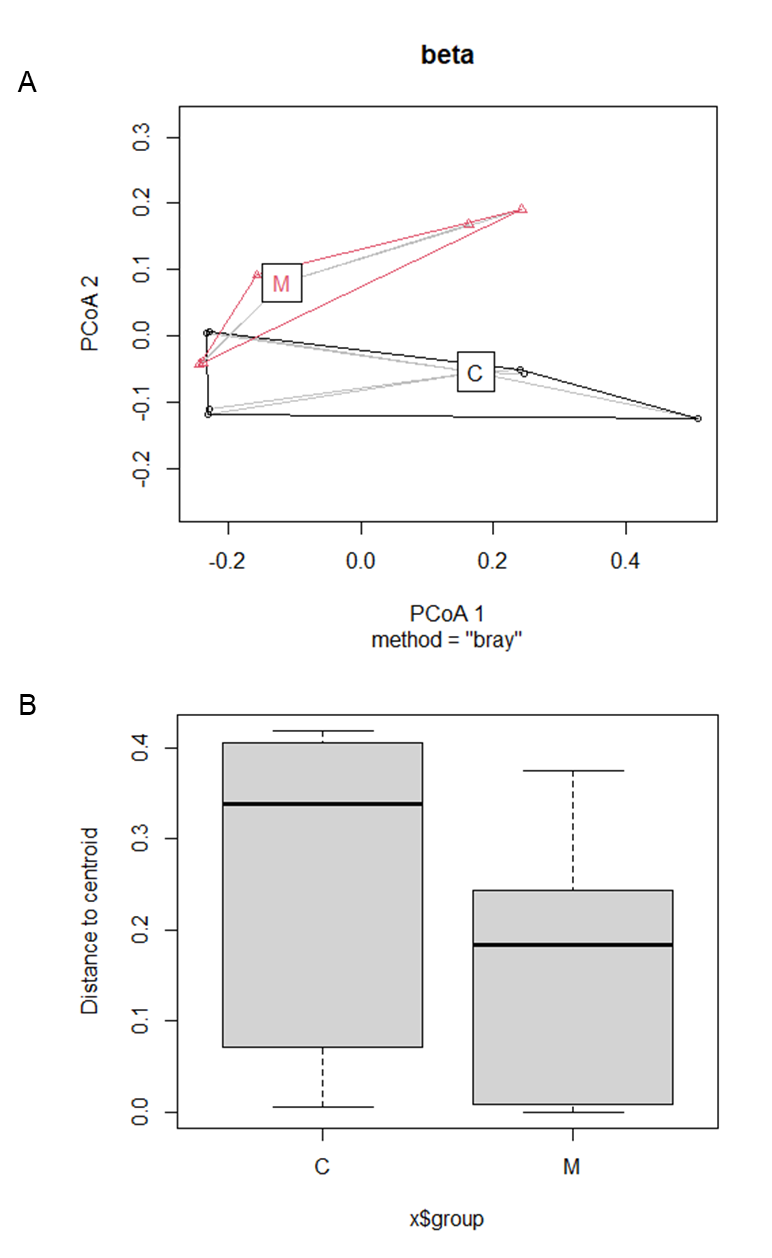
**

**Supplementary figure 1:** Permutational MANOVA (adonis) of the bacterial community of MBC PDO. A Principal coordinate analysis (PCoA) analysis of the bacterial community according to the dairy, B boxplot of the beta dispersion of samples from the two dairies.
